# Supplementary material for: Pyruvate Dehydrogenase Kinase 1 inhibition mediated oxidative phosphorylation enhancement in cartilage promotes osteoarthritis progression
Source: BMC Musculoskelet Disord. 2023 Jul 20;24:597. doi: 10.1186/s12891-023-06585-6 (PMC10357736; doi:10.1186/s12891-023-06585-6)
Supplement: Supplementary file 1 — Additional file 1. [file 12891_2023_6585_MOESM1_ESM.zip › BP.Gsea.1653623667859/GOBP_PIGMENT_GRANULE_LOCALIZATION.html]

Details for gene set GOBP\_PIGMENT\_GRANULE\_LOCALIZATION[GSEA]

|  || Dataset | 55457\_collapsed\_to\_symbols.cls.cls #OA\_versus\_normal.cls.cls #OA\_versus\_normal\_repos |
| Phenotype | cls.cls#OA\_versus\_normal\_repos |
| Upregulated in class | OA |
| GeneSet | GOBP\_PIGMENT\_GRANULE\_LOCALIZATION |
| Enrichment Score (ES) | 0.4548207 |
| Normalized Enrichment Score (NES) | 1.4517194 |
| Nominal p-value | 0.053658538 |
| FDR q-value | 0.42479146 |
| FWER p-Value | 1.0 |
Table: GSEA Results Summary

  

Fig 1: Enrichment plot: GOBP\_PIGMENT\_GRANULE\_LOCALIZATION      
 Profile of the Running ES Score & Positions of GeneSet Members on the Rank Ordered List

  

| SYMBOL | TITLE | RANK IN GENE LIST | RANK METRIC SCORE | RUNNING ES | CORE ENRICHMENT || 1 | MREG | melanoregulin [Source:HGNC Symbol;Acc:HGNC:25478] | 338 | 0.731 | 0.1338 | Yes |
| 2 | BBS4 | Bardet-Biedl syndrome 4 [Source:HGNC Symbol;Acc:HGNC:969] | 1404 | 0.442 | 0.1480 | Yes |
| 3 | RAB11A | "RAB11A, member RAS oncogene family [Source:HGNC Symbol;Acc:HGNC:9760]" | 1447 | 0.436 | 0.2402 | Yes |
| 4 | RAB27B | "RAB27B, member RAS oncogene family [Source:HGNC Symbol;Acc:HGNC:9767]" | 2296 | 0.327 | 0.2460 | Yes |
| 5 | ASIP | agouti signaling protein [Source:HGNC Symbol;Acc:HGNC:745] | 2596 | 0.291 | 0.2865 | Yes |
| 6 | GPR143 | G protein-coupled receptor 143 [Source:HGNC Symbol;Acc:HGNC:20145] | 2730 | 0.277 | 0.3369 | Yes |
| 7 | MLPH | melanophilin [Source:HGNC Symbol;Acc:HGNC:29643] | 2794 | 0.270 | 0.3911 | Yes |
| 8 | DCTN2 | dynactin subunit 2 [Source:HGNC Symbol;Acc:HGNC:2712] | 2888 | 0.262 | 0.4412 | Yes |
| 9 | MYO5A | myosin VA [Source:HGNC Symbol;Acc:HGNC:7602] | 3330 | 0.218 | 0.4548 | Yes |
| 10 | MKKS | MKKS centrosomal shuttling protein [Source:HGNC Symbol;Acc:HGNC:7108] | 4182 | 0.140 | 0.4193 | No |
| 11 | MYO7A | myosin VIIA [Source:HGNC Symbol;Acc:HGNC:7606] | 4808 | 0.087 | 0.3898 | No |
| 12 | VPS33B | VPS33B late endosome and lysosome associated [Source:HGNC Symbol;Acc:HGNC:12712] | 5297 | 0.048 | 0.3624 | No |
| 13 | SHROOM2 | shroom family member 2 [Source:HGNC Symbol;Acc:HGNC:630] | 6196 | -0.020 | 0.2971 | No |
| 14 | BBS7 | Bardet-Biedl syndrome 7 [Source:HGNC Symbol;Acc:HGNC:18758] | 6460 | -0.039 | 0.2852 | No |
| 15 | RAB17 | "RAB17, member RAS oncogene family [Source:HGNC Symbol;Acc:HGNC:16523]" | 6818 | -0.064 | 0.2716 | No |
| 16 | VPS33A | VPS33A core subunit of CORVET and HOPS complexes [Source:HGNC Symbol;Acc:HGNC:18179] | 7219 | -0.097 | 0.2618 | No |
| 17 | RAB11B | "RAB11B, member RAS oncogene family [Source:HGNC Symbol;Acc:HGNC:9761]" | 7227 | -0.097 | 0.2826 | No |
| 18 | CDH3 | cadherin 3 [Source:HGNC Symbol;Acc:HGNC:1762] | 7924 | -0.149 | 0.2612 | No |
| 19 | RAB1A | "RAB1A, member RAS oncogene family [Source:HGNC Symbol;Acc:HGNC:9758]" | 9604 | -0.284 | 0.1930 | No |
| 20 | RAB27A | "RAB27A, member RAS oncogene family [Source:HGNC Symbol;Acc:HGNC:9766]" | 9639 | -0.287 | 0.2532 | No |
Table: GSEA details [plain text format]

  

Fig 2: GOBP\_PIGMENT\_GRANULE\_LOCALIZATION      
 Blue-Pink O' Gram in the Space of the Analyzed GeneSet

  

Fig 3: GOBP\_PIGMENT\_GRANULE\_LOCALIZATION: Random ES distribution      
 Gene set null distribution of ES for **GOBP\_PIGMENT\_GRANULE\_LOCALIZATION**
